# Prenatal Alcohol Exposure Affects Progenitor Cell Numbers in Olfactory Bulbs and Dentate Gyrus of Vervet Monkeys

**DOI:** 10.3390/brainsci6040052

**Published:** 2016-10-27

**Authors:** Mark W. Burke, Alexey Inyatkin, Maurice Ptito, Frank R. Ervin, Roberta M. Palmour

**Affiliations:** 1 Department of Physiology and Biophysics, Howard University, Washington, DC 20059, USA; mark.burke@howard.edu; 2 Behavioural Science Foundation, St. Kitts, Saint Kitts and Nevis; ptito.maurice@gmail.com (M.P.); rmpskb@gmail.com (F.R.E.); 3 Departments of Human Genetics and Psychiatry, Faculty of Medicine, McGill University, Montréal, QC H3A 1A1, Canada; alexey.inyatkin@gmail.com; 4 School of Optometry and Department of Physiology, Université de Montréal, Montréal, QC H3C 3J7, Canada; 5 Laboratory of Neuropschiatry, University of Copenhagen, Copenhagen DK-2200, Denmark

**Keywords:** proliferating progenitor cells, stereology, fetal alcohol, olfactory bulb, hippocampus, non-human primate

## Abstract

Fetal alcohol exposure (FAE) alters hippocampal cell numbers in rodents and primates, and this may be due, in part, to a reduction in the number or migration of neuronal progenitor cells. The olfactory bulb exhibits substantial postnatal cellular proliferation and a rapid turnover of newly formed cells in the rostral migratory pathway, while production and migration of postnatal neurons into the dentate gyrus may be more complex. The relatively small size of the olfactory bulb, compared to the hippocampus, potentially makes this structure ideal for a rapid analysis. This study used the St. Kitts vervet monkey (*Chlorocebus sabeus*) to (1) investigate the normal developmental sequence of post-natal proliferation in the olfactory bulb and dentate gyrus and (2) determine the effects of naturalistic prenatal ethanol exposure on proliferation at three different ages (neonate, five months and two years). Using design-based stereology, we found an age-related decrease of actively proliferating cells in the olfactory bulb and dentate gyrus for both control and FAE groups. Furthermore, at the neonatal time point, the FAE group had fewer actively proliferating cells as compared to the control group. These data are unique with respect to fetal ethanol effects on progenitor proliferation in the primate brain and suggest that the olfactory bulb may be a useful structure for studies of cellular proliferation.

## 1. Introduction

Exposure to alcohol during fetal development is recognized as one of the most common preventable developmental disorders, with 10.2% of US women reporting alcohol consumption during pregnancy and 3.1% reporting binge drinking [1]. Heavy maternal drinking during the period of organogenesis results in a classic fetal alcohol syndrome that presents with a distinct form of craniofacial dysmorphologies and an array of neurodevelopmental deficits [2]. More commonly, individuals with fetal alcohol exposure exhibit developmental lag along with cognitive and behavioral impairments in the absence of severe facial anomalies. Fetal alcohol spectrum disorder (FASD) is a non-diagnostic umbrella term that encompasses the range of these impairments [3,4]. The actual prevalence of FASD remains elusive [5,6]. Estimates from South Africa place the incidence of FASD at about 5.9%–9.1% [7]; in North America, an incidence of 2%–5% has been reported [6,8], and in rural and isolated communities, the estimate may be as high as 26% [9,10,11]. The lifetime cost of FASD has been estimated to be up to $2 million per individual [12,13] in North America, but Swedish estimates place the societal cost of FAE between €76,000–110,000 per year per individual when factoring in direct (education, psychiatric disorders and alcohol/drug abuse) and indirect (e.g., reduced earning capacity) costs [5]. The overall cost of FASD to the individual and society is likely to be much higher [3,14,15].

Individuals affected with FASD display an array of pervasive neurobehavioral deficits affecting adaptive behavior and social competence [3,16,17,18], and these can lead to disrupted school experiences [16,19], drug and alcohol abuse [17,20] and legal issues [14,17]. Affected individuals often exhibit deficits in executive function [21,22], cognition [23] and attention [16,24,25,26,27,28]. Evidence from clinical and animal model studies suggests that the fronto-limbic system is acutely affected by FAE [29,30,31,32,33]. Within this context, the hippocampus is particularly vulnerable. Clinical imaging studies report reduction in hippocampal volume in FASD [34,35], and rodent studies have defined the third trimester equivalent as a critical period for ethanol-induced hippocampal neuron loss [36,37]. Our own data from a naturalistic non-human primate model of FAE demonstrate that, in addition to a 35% neuronal reduction of frontal cortex neurons [31], there is significant and pervasive loss of hippocampal volume and neuronal populations following moderate and voluntary maternal alcohol consumption during the third trimester [32]. 

The possibility that cellular paucity in the hippocampus and frontal cortex is a consequence of impaired neurogenesis is a topic of considerable interest [38,39,40,41,42]. Postnatal neurogenesis is widely accepted to occur in two regions of the mammalian brain, the subgranular zone (SGZ) of the dentate gyrus (DG) and the subventricular zone (SVZ) [43,44]. Although much of the stem cell discoveries have been made in rodent models, non-human primates have been shown to have an SVZ rostral migratory stream and proliferating population in the SGZ similar to rodents [44]. Neurogenesis in SVZ is followed by migration of new cells through the rostral migratory stream into the olfactory bulb (OB), whereas new neurons from DG migrate locally into the inner granule cell layer through radial migration [45]. Although the dentate subgranular (hilar) zone is a major site of postnatal neurogenesis [43,46,47], the OB is an attractive target for study as a potential reporter structure because of its relatively small size, rapid turnover of newly formed cells and functional role in social behavior [48,49,50,51].

Fetal alcohol exposure has been shown to have long-lasting implications for hippocampal neurogenesis in rodent models [40,52,53,54], and this may be related to the observed behavioral impairments [50]. Despite the role of the OB in social behavior and its neurogenic capacity [55], the effects of prenatal ethanol exposure on this structure have been inadequately explored in comparison to the hippocampus and other areas [56,57,58,59]. Earlier studies report that FAE in rodents reduces OB volume, mitral cell populations, number of neurospheres and granule cells [56,57,58,59]. It also impairs odor discrimination in both mice and human subjects [56,57]. In the present study, we tracked actively proliferating neural stem cells from both the olfactory bulb and subgranular zone of the dentate gyrus throughout the first two years of life in FAE offspring of vervet monkeys voluntarily consuming beverage alcohol in a social context.

## 2. Materials and Methods

### 2.1. Subjects

FAE and sucrose-control offspring derived from a large, pedigreed breeding colony of African green monkeys (*Chlorocebus sabeus*) housed in the laboratories of the Behavioural Science Foundation (BSF), St. Kitts. All animals in this colony are fed high protein primate chow (Harlan Teklad) and fresh fruit daily, and have water available ad libitum. The foundation holds a certificate of Good Animal Practice from the Canadian Council on Animal Care and an Office of Laboratory Animal Welfare (OLAW) registration (A5028). All studies described here were reviewed and approved by both the BSF (protocol 0403) and the McGill University (protocol 4627) Animal Care Committees.

Protocols for the development of FAE and sucrose control offspring have been previously described in some detail [32]. Briefly, alcohol-preferring adult females were identified and housed in small breeding groups with a same age alcohol-avoiding male. The groups were monitored behaviorally for reproductive activity, and the females were examined semi-weekly to identify gestational age. Alcohol (maximum 3.5 g alcohol/kg body weight) or isocaloric sucrose exposure was initiated at about embryonic day 95 (range 72–118) of the modal 165-day gestation for the species. To prevent recurrent cycles of withdrawal, alcohol was available 4 days per week (Monday, Tuesday, Thursday, Friday) and drinking water was always available to each animal. In order to record alcohol consumption accurately, both alcohol- and sucrose-exposed animals drank for this 4 h period in individual compartments contiguous to their social groups. Thus, animals did not experience anesthesia, gavage, social isolation or other stressors. During the period of alcohol exposure, semi-weekly saphenous blood samples were collected without anesthesia for the measurement of blood ethanol levels (alcohol dehydrogenase method, Sigma, St. Louis, MO, USA). Alcohol exposure was terminated at the time of parturition.

Olfactory lobes, hippocampus and frontal cortex were collected from 30 vervet offspring (23 males, 7 females) at the time of planned sacrifice (Table 1). Animals were deeply sedated with ketamine hydrochloride (10 mg/kg, intramuscular and euthanized with an overdose of sodium pentobarbital (25 mg/kg, intravenous) according to procedures approved by the American Veterinary Medical Association. Thereafter, animals were transcardially perfused with phosphate buffered saline (PBS-pH 7.4), followed by 4% paraformaldehyde solution in PBS (≈1 L). Brains were stereotaxically blocked into 1 cm slabs, extracted from the skull, and the olfactory bulbs were detached. These were then cryoprotected in 30% buffered sucrose for 48 h at 4 °C, frozen in isopentane at −65 °C and stored at −80 °C until further processing. Brain tissue underwent graded sucrose cryoprotection (10%, 20% and 30%) over the course of 7 days prior to freezing in isopentane at −65 °C [31,32].

### 2.2. Histology

Parallel series of coronal sections through the hippocampus (50 µm) and olfactory bulb (40 µm) were obtained for each animal with a spacing of 1/10 for the 5- and 24-month groups with 1/6 for the neonatal group. One complete series was Nissl-stained with cresyl-violet while other series were banked in antigen preserve [32]. We have previously reported neuronal number in frontal cortex and hippocampal regions [31,32] from many of the animals reported here.

### 2.3. Immunohistochemistry

A series throughout the extent of the hippocampus and olfactory bulb from each subject was processed for immunohistochemical staining of Ki-67, a cell-surface antigen present in proliferating cells, but absent in mature neurons [60], using a modification of the method of Eadie et al. [61]. This proliferation marker stains cells in all phases of the cell cycle after G0 and before final neuronal maturation [62]. Sections were washed five times in PBS to remove residual antigen preserve and then underwent antigen retrieval by incubation at 80 °C for 30 min in 10 mM citrate buffer (pH 6.0) containing 0.05% triton. After a 30 min cooling period at room temperature, the sections were washed 3 times in Tris-buffered saline (0.1 mM Tris, pH 7.5) and blocked for 1 h in 2% normal donkey serum (NDS) in Tris-buffered saline (TBS) containing 0.3% triton. After an additional set of TBS washes, sections were incubated overnight at 4 °C in rabbit anti-Ki-67 antibody (NeoMarkers: 1:500, Fremont, CA, USA) followed by secondary antibody (anti-rabbit 1:200; Jackson ImmunoResearch Laboratories Inc., West Grove, PA, USA). Next, the sections were incubated in ABC (Vector, Burlingame, CA, USA) for 2 h, and then processed with diaminobenzidine (DAB, Sigma, St. Louis, MO, USA) for visualization and mounted. Sections from both FAE and control subjects were immunostained without primary antibody as a negative control and there was a lack of background staining. For delineation purposes, sections were counterstained with the fluorescent nuclear stain, bisbenzidine (0.025%; Hoechst 33258, Sigma, St. Louis, MO, USA) for 20 min, dehydrated, cleared in xylenes and coverslipped with DPX mounting media (VWR, Radnor, PA, USA).

### 2.4. Stereology

**Figure 1:**
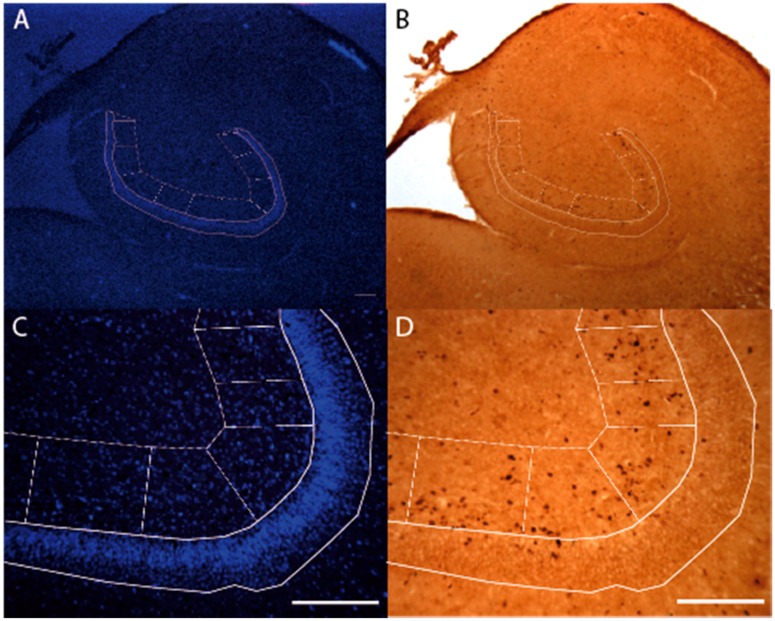
Rare event protocol method. The nuclear stain bisbenzidine was used to identify the granular layer and hilus/proliferation zone (**A**,**C**). Delineation of the granular layer and hilus were performed under a 10× objective with the bisbenzidine. The granular layer of the dentate gyrus was defined as a densely packed cell layer and the hilus as the 200 µm adjacent to this layer (**C**). Topography was then superimposed under light microscopy (**B**,**D**), and counts were performed using a 40× objective with the aid of BioQuant software (Version 10.10, BioQuant Life Science, Nashville, TN, USA). Scale bars (**A**,**B**) = 1 mm; (**C**,**D**) = 200 µm.

Design-based stereological estimation of total cell numbers (*N*) in a given region was calculated by the Equation (1):

(1) *N* = ssf^−1^ × asf^−1^ × tsf^−1^ × ∑*Q*−

where ssf is section sampling fraction, asf is area sampling fraction, tsf is the thickness-sampling fraction and ∑*Q*− is the number of cells counted within the dissection [63]. Due to the dispersed distribution of Ki-67+ cells in the hippocampal and olfactory regions, it would require a large number of disectors to obtain a valid population estimation using traditional design-based stereology. As a consequence, a modified rare event stereological protocol was used. The estimation of total immunopositive cells (*N*) was therefore calculated by the Equation (2):

(2) *N* = ssf^−1^ × 1 × 1 × ∑*Q*−

where the area of the counting frame and thickness relative to the sampled area and sampled thickness were the same (i.e., 100% of the outlined area and thickness were sampled), so that asf^−1^ and tsf^−1^ were each equal to 1. Delineation of the granular layer and hilus were performed with the bisbenzidine nuclear stain under a 10× objective and counts were acquired with BioQuant software (Version 10.10, BioQuant Life Science, Nashville, TN, USA), using a 40× objective on a light microscope (Figure 1). The granular layer of the dentate gyrus (DG) was defined as the densely packed cell layer and the hilus as the 200 µm adjacent to this layer. Sections equally spaced throughout the hippocampus were sampled for each individual and each immunopositive cell was counted within the outlined topography described above. The topography of the entire olfactory bulb was used as a single disector.

### 2.5. Statistical Analysis

Analysis of group (treatment, age) differences was performed using one-way ANOVA followed by a Fisher’s *t*-test implemented in StatView 5 (Version 5.0, SAS Institute, Cary, NC, USA). Factorial interactions utilized two-way ANOVA corrected for small sample sizes.

## 3. Results

### 3.1. Olfactory Bulb

**Figure 2:**
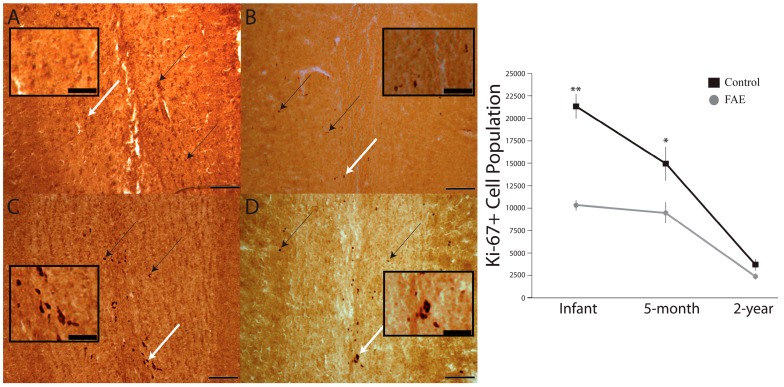
Olfactory bulb Ki67+ cells in fetal alcohol exposed (FAE) and control subjects of three developmental ages. Representative anatomical sections of olfactory bulb from control and FAE animals, at three different developmental ages. In the sections, Ki-67 positive cells can be observed as darkly stained cells (arrows). The photomicrographs displayed here were taken at 10×. (**A**) Neonatal control; (**B**) neonatal FAE; (**C**) five-month control; and (**D**) five-month FAE (Scale bar = 50 µm). In order to better display Ki-67+ cells, the regions corresponding to the white arrows were magnified in the inset for each image. Scale bar = 10 µm). The right hand panel depicts the progressive decline in proliferating cells in both treatment groups as a function of developmental age. There is a significant reduction of proliferating cells from infant to two years for both groups (FAE infant vs. two-year *p* < 0.01; Control infant vs. five-month vs. two-year *p* < 0.01). ** *p* < 0.001 and * *p* < 0.05 FAE vs. control; error bars are standard errors.

Representative anatomical sections from control and FAE animals, at three different developmental ages, are presented in Figure 2. In neonates and at five months, olfactory lobe Ki-67+ cells were more plentiful in control animals than in FAE animals, with the maximum estimated population (*n* = 23,560) occurring in the neonate control group and the minimum estimated population (*n* = 2100) in the two-year-old FAE group. There were main effects of age (*F*_2,14_ = 88.77, *p* < 0.0001) and ethanol exposure (*F*_1,14_ = 49.63, *p* < 0.0001), with the average number of Ki-67 positive cells being higher in sucrose controls than in the FAE offspring at the infant and five-month time points (*p* < 0.001 and *p* < 0.05, respectively). Finally, there was a significant age by treatment interaction effect (*F*_2,14_ = 11.59, *p* = 0.0011). The developmental nature of these effects is emphasized by the age-dependent decrease in Ki-67+ cells for both control and FAE groups. Paradoxically, the age-related rate of decrease was higher in control animals than it was for FAE offspring.

### 3.2. Dentate Gyrus

**Figure 3:**
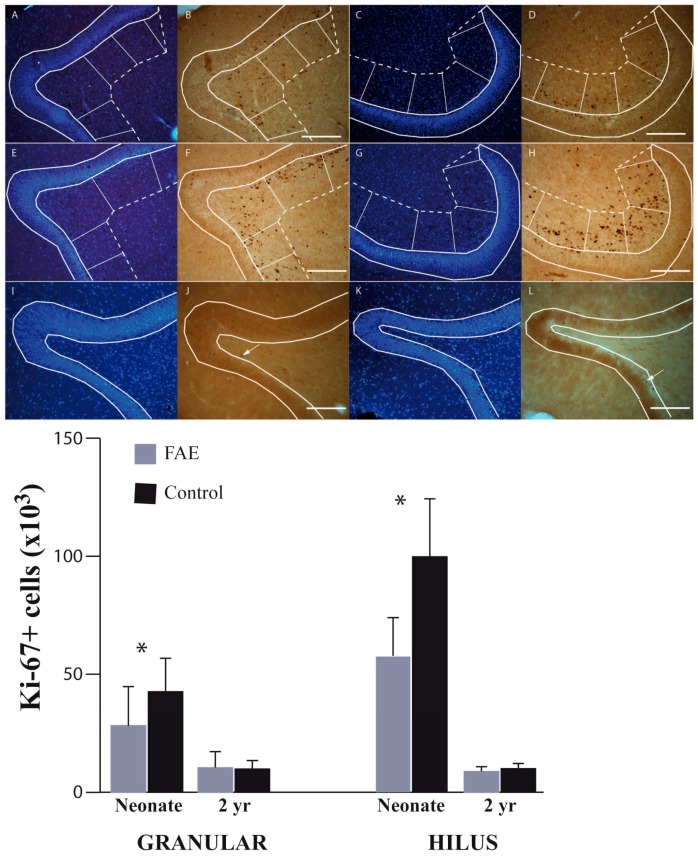
Dentate gyrus Ki-67+ cells in FAE and control subjects of two developmental ages. Photomicrographic images of the dentate gyrus (DG) and hilus regions taken with a 10× objective. Bisbenzidine was used to identify the granular layer and hilus/proliferation zone (**A**,**C**,**E**,**G**,**I**,**K**); at the neonatal time point, FAE subjects (**B**,**D**) show a significant reduction in actively proliferating cells evidenced by Ki-67 immunoreactivity, as compared to control subjects (**F**,**H**); and at the two-year old time point, the number of Ki-7+ cells is reduced in both FAE (**J**) and control subjects (**I**); and scale bar = 200 µm. Neonatal alcohol-exposed animals have fewer Ki-67+ cells in both the DG and hilus when compared to control animals, but this is only statistically significant for the hilus. Between the neonatal period and age 2 there was significant reduction in Ki-67+ cells for both groups, but no difference between groups. * *p* < 0.05; error bars are standard errors.

We report here a comparison of the neonatal (1–35 days) and the two-year-old hippocampus DG from normal vervets and those exposed to known levels of alcohol in utero (Figure 3). The 2-year time-point is well after the postnatal brain growth spurt and subsequent pruning, but well before the adolescent pruning period. When dentate hilus and granular regions were combined, there were 43% fewer Ki-67+ cells in neonatal FAE subjects as compared to control subjects. At two years of age, the effect of treatment on total Ki-67+ cell population was no longer significant (*p* = 0.973). When examining the effects of FAE on granular versus hilus region of the DG, the Ki-67+ cell population in the hilus showed a greater sensitivity to FAE than that of the granular region. There were significant main effects of group (*F*_1,13_ = 6.820, *p* = 0.0215), age (*F*_1,13_ = 53.355, *p* < 0.0001) and a significant age by group interaction (*F*_1,13_ = 5.972, *p* = 0.0296) within the hilus region. There were significantly fewer Ki-67+ cells in the FAE subjects compared to controls (*p* = 0.003) in the neonatal timepoint. By age 2, actively proliferating progenitor cells had diminished substantially in both groups. As with the OB, the proportional loss was greater in the control group, so that cell numbers no longer differ significantly between groups.

## 4. Discussion

In previous reports on this naturalistic model of maternal ethanol consumption by an Old World monkey evolutionarily close to man, we showed systematic and quantitative brain changes in response to levels of alcohol consumption frequently encountered in a social context [31,32,64]. In the present report, we extend our observations to include the olfactory bulb and dentate gyrus, two regions that are important in adult neurogenesis. Using design-based stereology, we show that animals exposed to beverage alcohol during the last trimester of gestation have lower numbers of Ki-67 positive cells in both OB and DG, as compared to sucrose control individuals. This finding was most obvious perinatally, when FAE groups had approximately 50% of the cell counts seen in sucrose controls in both OB and DG. As expected, Ki-67 positive cell counts were reduced in both groups and all regions as a function of developmental age, but the rate of decrease was proportionally greater in the control group so that absolute cell numbers at two years of age were much closer between groups. There is no published data concerning proliferating cell numbers in the OB of non-human primates, but the level of Ki-67+ cells in the dentate gyrus of control animals at birth and two years of age is consistent with previous non-human primate studies [65]. Because of the focus on proliferating cells, a limitation of this study is that we were not able to determine the fate (whether neuron or glia) of the progenitor cells.

In primates, OB neurogenesis and a portion of DG neurogenesis are fed, through different migratory pathways, by proliferating cells from the subventricular zone (SVZ) lining the lateral ventricle [37,48,66,67,68]. Post-natal OB neurogenesis has been shown to be related to neurogenesis in SVZ from two months to 24 months of age in mice [69], and this pattern might also characterize striatal neurogenesis in the adult rat brain [70]. The developmental trajectories of proliferating stem cells shown in this study are quite similar in OB and DG, both in sucrose control and FAE animals.

Although we have not conducted interventional studies in this model, these data suggest that changes in the proliferating population in OB could be a rapid and efficient indicator of efficacy. This notion is strengthened by murine studies showing that the OB is differentially responsive to ethanol toxicity in a full-gestation exposure model [56].

The hypothesis that pre- and perinatal ethanol damage is related to inhibition of prenatal neurogenesis has been studied by many groups (review: [38]) but has not reliably been supported. This relates, at least in part, to different species, different models of ethanol exposure and different developmental time points. Quite importantly, however, it may also be related to the specific measure of prenatal neuronal proliferation that is used. It is generally acknowledged that transient amplifying progenitors (typically identified by some marker of mitosis such as 5-Bromo-2′-deoxyuridine-BrdU) progress through various early stages of maturation (marked by Ki-67 and doublecortin-DCX positive cells) before becoming mature (neuronal nuclear protein-Neu-N positive) neurons. Several recent rodent studies [71,72,73,74], including one [74] that tracked the development of post-natally born hippocampal neurons from birth through maturation, allege that there is no significant difference, under standard housing conditions, of BRDU+ DG cells between animals expressed to moderate doses of alcohol during gestation and sucrose controls. It is well documented, however, that voluntary exercise in rodents (both FAE and control) increases postnatal neuronal proliferation as well as ameliorating learning and spatial memory deficits associated with hippocampal dysfunction [38,71,72,73,74] and increasing evidence that environmental complexity is an important factor in retention of these newly born neurons in sucrose controls, but not in FAE animals [73,74,75]. Detailed analysis [74] suggests that the cellular diminution in FAE animals is not apparent at cell birth, as measured by BrdU labelling, and becomes apparent during maturational stages that are later than those marked by Ki-67. These results are consistent with some previous observations that did not use such a fine-grained analysis [52,54]. Given this conceptualization, the data reported here suggests that the effects of prenatal ethanol on proliferating cell populations may be apparent at an earlier developmental time in non-human primates than it is in rodents, or, alternatively, there might be some diminution of cell number between BRDU and Ki-67+ stages. A full characterization of the process will require further experimentation, with birth-dating of cells as well as documentation of maturational stages with markers such as DCX, nestin and NeuN at a range of developmental points throughout the first few years of life.

For those interested in the possibility of cellular therapeutics, the identity of processes and metabolites involved in progenitor cell maturation necessarily becomes important [71,75,76]. There are many leads, but few directed or hypothesis-testing studies. Fetal alcohol exposure alters the expression of proteins involved in cell growth, proliferation, survival and differentiation in ex vivo model systems [77,78]. Prenatal ethanol exposure has also been proposed to disrupt the developmental cycle of normal stem cells [79,80]. Specifically, ethanol is thought to induce cyclin D1 expression, promote premature S-phase entry and enable disjointed DNA synthesis with increased apoptosis [79]. The possibility that these effects act indirectly, through the neuronal environment, is a topic of current interest [54,74] as is the possibility that some components of maturational inhibition may be epigenetic [81] or even trans-generational [42].

From a clinical perspective, the good news is that many progenitor cells seem to be alive and in proper position for functioning if their maturation could be promoted. There are a number of promising (and relatively simple) therapeutic strategies to be explored. What is now required is to determine the effect of these strategies on neuronal maturation and retention.

## 5. Conclusions

In summary, we have shown both age-and drug-related effects on the number of maturing neural progenitor cells in the olfactory bulb and dentate gyrus of the vervet monkey. Age-related decreases were quite consistent with those shown previously in rodents and in other primates, including man. Although some previous studies have suggested that prenatal ethanol exposure might decrease, or even obliterate, the neural progenitor cell population, to our knowledge, this is the first demonstration of an in vivo prenatal ethanol effect in the proliferative zone of the olfactory bulb, and the first demonstration of age-by-treatment effect on Ki-67 positive cell numbers in non-human primates. These data suggest a point of entry for potential restorative interventions, and also suggest that the olfactory bulb might be a useful target for assessing the efficacy of pharmacological and other interventional studies.

## Figures and Tables

**Table 1:** Characteristics of subjects. Systematic sections through the olfactory bulb (OB) and dentate gyrus (DG) were available for a subset of subjects.

Animal	Sex	Region	Day Alc Started	Drinking Days	Av Alc (g/kg/day)	Average BEC	Age at Sacrifice
O1808-1	m	DG	131	19	3.23	135	12 days
O2898-5	m	DG	96	40	3.19	108	15 days
O3245-3	f	DG	66	56	3.39	129	3 days
O3307-3	f	OB/DG	94	40	3.17	132	35 days
O5011-4	m	OB/DG	82	42	1.97	72	15 days
O2780-5	m	OB	93	36	3	118	10 days
O5232-2	m	DG	77	0	sucrose	0	9 days
O6228-1	m	DG	90	0	sucrose	0	18 days
O6332-1	f	DG	121	0	sucrose	0	12 days
O6692-1	m	DG	115	0	sucrose	0	30 days
O6712-1	m	DG	72	0	sucrose	0	1 days
O3295-5	f	OB	86	0	sucrose	0	15 days
O6172-1	m	OB	125	0	sucrose	0	10 days
O5329-1	m	OB	109	0	sucrose	0	6 days
O3065-8	m	OB	94	41	2.98	125	5 months
O5399-1	m	OB	106	35	2.48	101	5 months
O5859-1	m	OB	127	22	2.24	75	5 months
O5173-2	f	OB	118	0	sucrose	0	5 months
O6503-1	m	OB	103	0	sucrose	0	5 months
O9184-4-2	m	OB	108	0	sucrose	0	5 months
O3327-1	m	DG	115	30	2.98	120	21 months
O5011-3	m	OB	77	48	2.81	118	19 months
O3295-3	m	OB/DG	75	51	2.91	97	22 months
O3327-2	m	OB/DG	113	31	2.77	104	22 months
O2780-6	m	OB	113	30	2.98	95	26 months
O5603-2	m	DG	112	0	sucrose	0	22 months
O3060-5	m	OB/DG	116	0	sucrose	0	21 months
O5151-1	m	OB/DG	73	0	sucrose	0	19 months

The day alcohol or sucrose was started indicates the gestational day. Average ethanol (g/kg) indicates the amount ingested by the dam on a daily basis. Abbreviations: m = male, f = female, Alc = alcohol, Av = average, BEC = blood ethanol concentration (mg/dL).
